# Impact of neuroendocrine morphology on cancer outcomes and stage at diagnosis: a UK nationwide cohort study 2013–2015

**DOI:** 10.1038/s41416-019-0606-3

**Published:** 2019-10-25

**Authors:** Tracey S. E. Genus, Catherine Bouvier, Kwok F. Wong, Rajaventhan Srirajaskanthan, Brian A. Rous, Denis C. Talbot, Juan W. Valle, Mohid Khan, Neil Pearce, Mona Elshafie, Nicholas S. Reed, Eileen Morgan, Andrew Deas, Ceri White, Dyfed Huws, John Ramage

**Affiliations:** 1 Neuroendocrine Tumour Patients Foundation, Leamington Spa, UK; 2 National Cancer Registration and Analysis Service, Public Health England, Birmingham, UK; 3 Department of Gastroenterology, King’s College Hospital, London, UK; 4 ENETS Centre of Excellence, Neuroendocrine Tumour Unit, King’s College Hospital, London, UK; 5 National Cancer Registration and Analysis Service, Public Health England, Cambridge, UK; 6 ENETS Centre of Excellence, Oxford University Hospitals Trust, Oxford, UK; 7 Department of Medical Oncology, The Christie NHS Foundation Trust, Manchester, UK; 8 Department of Gastroenterology, University Hospital of Wales, Cardiff & Vale University Hospital Board, Cardiff, UK; 9 Department of Hepatobiliary and Pancreatic Surgery, University Hospital Southampton NHS Foundation Trust, Southampton, UK; 10 Department of Cellular Pathology, University Hospitals Birmingham NHS Foundation Trust, Birmingham, UK; 11 Beatson Oncology Centre, Gartnavel General Hospital, Glasgow, UK; 12 Northern Ireland Cancer Registry, Centre for Public Health, Queen’s University Belfast, Belfast, UK; 13 Information Services Division, NHS National Services Scotland, Edinburgh, UK; 14 Welsh Cancer Intelligence & Surveillance Unit, Public Health Wales, Cardiff, UK

**Keywords:** Cancer epidemiology, Cancer epidemiology, Gastrointestinal cancer, Neuroendocrine cancer, Neuroendocrine cancer

## Abstract

**Background:**

The diagnosis of neuroendocrine neoplasms (NENs) is often delayed. This first UK population-based epidemiological study of NENs compares outcomes with non-NENs to identify any inequalities.

**Methods:**

Age-standardised incidence rate (ASR), 1-year overall survival, hazard ratios and standardised mortality rates (SMRs) were calculated for all malignant NENs diagnosed 2013–2015 from UK national Public Health records. Comparison with non-NENs assessed 1-year overall survival (1YS) and association between diagnosis at stage IV and morphology.

**Results:**

A total of 15,222 NENs were identified, with an ASR (2013–2015 combined) of 8.6 per 100,000 (95% CI 8.5–8.7); 4.6 per 100 000 (95% CI, 4.5–4.7) for gastro-entero-pancreatic (GEP) NENs. The 1YS was 75% (95% CI, 73.9–75.4) varying significantly by sex. Site and morphology were prognostic. NENs (predominantly small cell carcinomas) in the oesophagus, bladder, prostate, and female reproductive organs had a poorer outcome and were three times more likely to be diagnosed at stage IV than non-NENs.

**Conclusion:**

Advanced stage at diagnosis with significantly poorer outcomes of some NENs compared with non-NENs at the same anatomical site, highlight the need for improved access to specialist services and targeted service improvement.

## Background

Neuroendocrine neoplasms (NENs) occur throughout the body, the most common sites including pulmonary, digestive and skin. NENs range from well-differentiated neuroendocrine tumours (NETs) to poorly differentiated carcinomas (NECs, small- and large-cell type) (World Health Organisation [WHO] Classification 2017) having varying potential, low grade and indolent to high grade and aggressive. The heterogeneous clinical presentation and biology of NENs cause significant challenges in diagnosis and management,^1^ with NENs often misdiagnosed, or diagnosis frequently delayed.^2^

The UK cancer registries in England, Scotland, Wales and Northern Ireland systematically collect high quality histologically confirmed and validated data on all malignant cancers diagnosed. However, previous coding systems have made comprehensive isolation of NEN data for epidemiological studies impossible. Registration in the ICD-O-3 (combined topology and morphology) coding system for cases diagnosed from 2013 onward,^3^ and TNM staging of GEP-NENs from 2010^4^ have now allowed the first descriptive epidemiological study of NENs diagnosed in the UK, and the first analysis by TNM stage.

The aim of this study was to determine the epidemiology of NENs diagnosed in the UK, and to ascertain whether there are any disparities in outcomes between NENs and non-NENs at the same anatomical site.

## Methods

### Study population

A comprehensive population-based cohort study was undertaken for all persons diagnosed with NENs in the UK between 1 January 2013 and 31 December 2015 with follow-up to the week commencing 12 January 2017, using patient-level data collected by Public Health England National Cancer Registration and Analysis Service (PHE NCRAS), and pseudo-anonymised data from NHS National Services Scotland, Public Health Wales Welsh Cancer Intelligence & Surveillance Unit and Public Health Agency Queen’s University Belfast. These registries had coverage of a 2015 mid-year total population of 65,110,000. Socioeconomic status (not available for Scotland) was measured by deprivation quintiles based on: area deprivation for England and N. Ireland, produced by the Ministry of Housing, Communities and Local Government (formerly the Department for Communities and Local Government); and aspects of deprivation experienced in income for Wales, produced by the Welsh Government using the Welsh Index of Multiple Deprivation. English data for non-NENs was also available for comparison.

### Tumour classification

NENs were defined by the WHO 2015 classification excluding diffuse pulmonary neuroendocrine hyperplasia. Tumours occurring at all anatomical sites between C00 and C80 according to the 10th edition of the WHO International Classification of Disease (ICD-10) codes were included, and morphology codes included 8013 (excluding lung [C34 and C78]), 8041–8045 (excluding lung), 8150–8158, 8240–8247, 8249 and 9091 according to the WHO International Classification of Diseases for Oncology, 3rd Edition (ICD-O-3). All were behaviour code 3 (malignant), 6 (metastatic) or 9 (malignant, uncertain whether primary or metastatic).

Tumours were staged according to the American Joint Committee on Cancer (AJCC) TNM staging for NENs^5^ and the European Neuroendocrine Tumor Society (ENETS) recommendations for the staging of GEP-NENs^6^ and graded using the ENETS grading system (WHO 2010) for GEP-NENs,^7–9^ or according to pathological grading of differentiation for other sites. Ki-67 status was not uniformly captured and is not recognised yet in many organ systems. Due to this being a registry study, we were unable to revisit each tissue specimen to acquire it, most grades were therefore based on a morphology of “poorly differentiated” or “well-differentiated” so grade 1 and grade 2 were combined to reduce inaccuracies. Tumour grades were classified: grade 1 and grade 2 combined—NET G1/G2 (functioning tumours including insulinomas, glucagonomas etc. with morphology codes 8150–8153, 8155–8158 and tumours with morphology codes 8240–8242, 8249, and 9091); grade 3—NEC G3 (large cell carcinoma, small cell carcinoma, combined small cell carcinoma, neuroendocrine carcinoma NOS with morphology codes 8013, 8041–8045, and 8246); Mixed Adeno-Neuroendocrine Carcinoma (MANEC) (Now known as ‘goblet cell adenocarcinomas’) goblet cell carcinoid, mixed adenoneuroendocrine carcinoma, tubular adenocarcinoid tumour with morphology codes 8243–8245); and “other” (pancreatic endocrine tumour malignant, mixed pancreatic endocrine and exocrine tumour malignant, and Merkel cell carcinoma morphology codes 8150, 8154 and 8247, respectively).

### Statistical analysis

All analyses were performed using STATA/MP 15.1 for Windows software program. ASRs for the UK 2013–2015 combined, UK 2013–2015 annual and 2001–2015 time series (England only) were calculated using ICD-O-2 codes for diagnoses between 2001 and 2012 (morphology codes 8013 [large cell neuroendocrine carcinoma], and 8249 [atypical carcinoid tumour] were not available), 1971–2015 mid-year population estimates Office of National Statistics (ONS) data,^10^ and the 2013 European standard population. Of the cohort, 15,106 persons were included in survival and mortality analyses, excluding those with a death certificate only registration and including the first tumour in those with multiple tumours, or the tumour with known stage if multiple tumours had the same diagnosis date. 1-year overall survival (1YS) estimates (also for non-NENs) were made using the Kaplan–Meier method with log rank tests. Multivariable survival analysis using Cox (proportional hazards) regression and likelihood ratios were performed with the model adjusting for clinically relevant independent variables sex, age, stage, site, morphology, grade, deprivation and year of diagnosis (all assessed for significant association with overall survival at *p* < 0.05). Standardised mortality rates, for those diagnosed between 1 January 2013 and 31 December 2015, for all-cause mortality over the disease duration, adjusting for explanatory variables age, sex and calendar period, were calculated by means of the life-table approach using ONS death registrations, including persons aged 0–100 years.^11,12^ Multiple logistic regression estimated the odds ratios (OR) and 95% confidence intervals (CI) for association of diagnosis at stage IV with morphology (NEN or non-NEN), for all people diagnosed with cancer between 2013 and 2015 in England, adjusting for sex and age. Vital status on 1 January 2017 was acquired from ONS.

## Results

### Patients

Overall, 15,145 persons were diagnosed with 15,222 NENs between 2013 and 2015, of which 7,640 (50.4%) were female. The average age at diagnosis was 65–69 years old with variation between sites (Table 1). The median follow-up time was 19.6 months (range 0–49.2 months) and 24.8% (3,766) died within one year of diagnosis.

**Table 1:** Demographic and clinical characteristics table

Morphology sparklines: represents the distribution of morphologies by site, the darkest column the most frequent morphology

*C* neuroendocrine (carcinoid) tumour, *S* small cell neuroendocrine carcinoma, *N* neuroendocrine carcinoma, *M* merkel cell carcinoma, *O* other, *NEC G3* neuroendocrine carcinoma grade 3, *n* number of patients, *N/A* not applicable

^a^Proportion of those with known stage

^b^Females

^c^Non-melanoma skin cancers are not currently staged

### Tumours

The most common primary tumour sites were pulmonary 20%, small intestine 13%, appendix 12%, pancreas 9%, skin 7%, colon and caecum 5%, stomach 5%, rectum 4%, bladder 3% and oesophagus 3%. Stage distribution was 37% localised (18% stage I, 9% stage II and 11% stage III), 23% metastasised (stage IV) and 39% unknown. Around 42% of the neoplasms were NETs, 28% neuroendocrine carcinomas and 9% small cell carcinomas. Nearly half of the tumours (48%) were low grade (grade 1 or 2) and over a third (35%) high grade (grade 3), the remaining tumours were MANEC (3.5%) and other (13%). Tumour breakdown by proportion of stage IV, grade 3 and by morphology for the most common sites is shown in Table 1.

### Incidence

The UK ASR was 8.6 per 100,000 (2013–2015 combined), 8.1 in females and 9.1 in males; 4.6 for GEP-NENs overall, the second most common gastrointestinal tumour.^13^ Incidence by other sites are listed in Table 2.

**Table 2:** NEN incidence, 1-year overall survival probability, multivariable analysis and mortality in the UK (2013–2015 combined)

Variables		Tumour count	Incidence	Survival^a^		Mortality^a^	
			Age-standardised rate (ASR) (CI) (per 100,000 persons)	1-year overall survival (Kaplan-–Meier) (CI)	Cox proportional hazards regression multi-variable (CI)	Observed/ expected deaths	Standardised mortality ratio (SMR) (CI)
All		15,222	8.6 (8.46–8.74)	74.7 (73.9–75.4)	–	5233/1442.6	3.6 (3.5–3.7)
Sex	1	7538	9.14 (8.93–9.35)	71.3 (70.3–72.4)	1 (reference)	2884/821.5	3.5 (3.4–3.6)
	2	7684	8.06 (7.88–8.24)	77.9 (76.9–78.8)	0.9 (0.8–0.9)	2349/621.1	3.8 (3.6–3.9)
Age	0–54	3668	1.88 (1.82–1.94)	90.2 (89.2–91.2)	0.5 (0.5–0.6)	545/156.6	3.5 (3.2–3.8)
	55–64	2865	1.62 (1.56–1.68)	78.7 (77.2–80.2)	0.8 (0.7–0.8)	553/56.6	9.8 (9–10.6)
	65–74	4302	2.47 (2.39–2.54)	72.5 (71.1–73.9)	1 (reference)	1284/223.8	5.7 (5.4–6.1)
	75+	4387	2.62 (2.54–2.7)	60.5 (58.9–61.9)	1.5 (1.4–1.6)	2851/1005.7	2.8 (2.7–2.9)
Deprivation	1- least deprived	2861	1.65 (1.59–1.71)	77.3 (75.7–78.8)	1 (reference)	920/317.3	2.9 (2.7–3.1)
	2	2915	1.67 (1.61–1.73)	75.3 (73.7–76.8)	1.1 (1–1.2)	990/303.6	3.3 (3.1–3.5)
	3	2835	1.62 (1.56–1.68)	75.9 (74.3–77.5)	1.1 (1–1.2)	978/278.4	3.5 (3.3–3.7)
	4	2640	1.51 (1.45–1.57)	73.1 (71.3–74.7)	1.2 (1.1–1.3)	946/240.1	3.9 (3.7–4.2)
	5- most deprived	2465	1.39 (1.34–1.45)	73.3 (71.5–75.1)	1.3 (1.2–1.4)	855/184.6	4.6 (4.3–5)
Diagnosis Year	2013	4895	8.41 (8.18–8.66)	74.1 (72.8–75.3)	1 (reference)	1997/622.7	3.2 (3.1–3.4)
	2014	5126	8.67 (8.43–8.92)	74.4 (73.2–75.6)	1 (0.9–1.1)	1799/487.5	3.7 (3.5–3.9)
	2015	5201	8.68 (8.44–8.92)	75.5 (74.3–76.7)	1.1 (1–1.1)	1437/332.5	4.3 (4.1–4.6)
Site	Appendix	1807	0.95 (0.9–0.99)	96.4 (95.4–97.2)	1 (reference)	122/89.1	1.4 (1.1–1.6)
	Breast	107	0.06 (0.05–0.07)	84.8 (76.3–90.4)	1.5 (1–2.3)	25/13.4	1.9 (1.3–2.8)
	Bladder	500	0.31 (0.28–0.34)	51.2 (46.6–55.7)	2.4 (1.9–3.1)	323/59.9	5.4 (4.8–6)
	Colon and caecum	734	0.41 (0.38–0.44)	72 (68.5–75.2)	2.4 (1.9–3.1)	249/62.9	4 (3.5–4.5)
	Female reproductive organs (incl.C51–C57)	378	0.2 (0.18–0.22)	69.2 (64.1–73.7)	2.8 (2.2–3.7)	158/12.8	12.3 (10.6–14.4)
	Oesophagus	470	0.26 (0.24–0.29)	35.5 (31–40.1)	3.4 (2.7–4.4)	366/26.1	14 (12.7–15.5)
	Pancreas	1415	0.8 (0.76–0.84)	80.6 (78.4–82.6)	2 (1.6–2.5)	421/112.9	3.7 (3.4–4.1)
	Prostate	137	0.08 (0.07–0.10)	31.5 (23.6–39.8)	2.6 (1.9–3.5)	114/8.6	13.2 (11–15.9)
	Pulmonary	2989	1.68 (1.62–1.74)	73.7 (72–75.3)	2.9 (2.3–3.6)	1019/232	4.4 (4.1–4.7)
	Rectum	622	0.32 (0.29–0.35)	81 (77.6–83.9)	2.7 (2–3.5)	151/42.6	3.5 (3–4.2)
	Skin	1044	0.62 (0.58–0.66)	74.3 (71.5–76.9)	2.1 (1.5–3)	460/292.2	1.6 (1.4–1.7)
	Small Intestine^b^	2054	1.17 (1.12–1.23)	89.6 (88.1–90.8)	1.3 (1–1.7)	157/83.7	1.9 (1.6–2.2)
	Ileum	1,024	0.53 (0.5–0.57)	92.8 (91–94.2)	ND	149/120.4	1.2 (1.0–1.4)
	Duodenum	316	0.18 (0.16–0.2)	87.3 (83–90.6)	ND	54/34.8	1.5 (1.2–2.0)
	Jejunum	40	0.02 (0.02–0.03)	89.4 (74.1–95.9)	ND	8/5.7	1.4 (0.7–2.8)
	Stomach	749	0.43 (0.4–0.46)	74.1 (70.8–77.2)	2.4 (1.9–3.1)	240/69.9	3.4 (3–3.9)
Stage	I	2691	1.48 (1.42–1.53)	96.1 (95.3–96.8)	1 (reference)	208/234.4	0.9 (0.8–1)
	II	1412	0.79 (0.75–0.84)	87.6 (85.7–89.2)	2.1 (1.8–2.6)	283/150.5	1.9 (1.7–2.1)
	III	1619	0.92 (0.87–0.97)	81.7 (79.6–83.5)	3.2 (2.7–3.8)	477/161.1	3 (2.7–3.2)
	IV	3493	2.01 (1.94–2.08)	48.8 (47–50.5)	8.2 (7.1–9.5)	2141/217.1	9.9 (9.5–10.3)
	Unknown	6007	3.39 (3.3–3.48)	74.5 (73.3–75.6)	3.6 (3.1–4.2)	2124/679.6	3.1 (3–3.3)
Grade	MANEC^c^	537	0.3 (0.27–0.33)	84.3 (80.8–87.2)	0.5 (0.1–3.6)	136/42.3	3.2 (2.7–3.8)
	NEC G3	5413	3.11 (3.03–3.2)	51.2 (49.8–52.6)	0.9 (0.8–1)	3225/409.3	7.9 (7.6–8.2)
	NET G1/G2	7316	4.04 (3.95–4.14)	92.7 (92.1–93.3)	0.4 (0.2–1.1)	940/648.1	1.5 (1.4–1.5)
	Other^d^	1956	1.14 (1.08–1.19)	65.8 (63.5–67.9)	1 (reference)	932/343	2.7 (2.5–2.9)
Morphology	Atypical carcinoid tumour	810	0.45 (0.42–0.48)	89.8 (87.5–91.7)	0.6 (0.2–1.6)	162/75.1	2.2 (1.8–2.5)
	Neuroendocrine tumour	6462	3.57 (3.48–3.66)	93.1 (92.4–93.7)	0.5 (0.2–1.2)	771/570.7	1.4 (1.3–1.5)
	Combined small cell carcinoma	285	0.17 (0.15–0.19)	54.8 (48.5–60.6)	1 (reference)	175/26.2	6.7 (5.8–7.7)
	Goblet cell carcinoid	335	0.18 (0.17–0.21)	92.1 (88.6–94.5)	1.1 (0.1–8.7)	53/26.9	2 (1.5–2.6)
	Large cell neuroendocrine carcinoma	221	0.13 (0.11–0.14)	43.1 (36.1–49.8)	1.3 (1.1–1.7)	151/14.3	10.6 (9–12.4)
	Merkel cell carcinoma	1057	0.63 (0.59–0.67)	73.4 (70.6–76)	0.5 (0.4–0.7)	469/288.2	1.6 (1.5–1.8)
	Mixed adeno-neuroendocrine carcinoma	188	0.11 (0.09–0.12)	68.6 (61.2–74.9)	1.6 (0.2–12.6)	82/14.8	5.5 (4.4–6.9)
	Neuroendocrine carcinoma NOS	4344	2.48 (2.41–2.56)	55.1 (53.5–56.6)	0.9 (0.7–1)	2362/326.9	7.2 (6.9–7.5)
	Other^e^	118	0.06 (0.05–0.08)	89.4 (82.1–93.8)	0.5 (0.3–0.8)	26/7.4	3.5 (2.4–5.1)
	Small cell carcinoma	1402	0.82 (0.77–0.86)	41.4 (38.7–44.1)	1.3 (1.1–1.5)	982/92	10.7 (10–11.4)

Socioeconomic status was not available for Scotland, so deprivation for Scotland was not included see Methods

Only the most common sites are listed, for additional sites see Supplementary Table 1

*ND* not determined

^a^Excluding “death certificate only” registration i.e. date of diagnosis = date of death

^b^Small intestine includes: C17.0—duodenum; C17.1—jejunum; C17.2—ileum; C17.3—Meckel’s diverticulum; C17.8; overlapping lesion of small intestine; C17.9 small intestine, unspecified

^c^MANEC  includes: goblet cell carcinoid; mixed adenoneuroendocrine carcinoma; tubular adenocarcinoid tumour

^d^Other grade includes pathology-graded: pancreatic endocrine tumour malignant; mixed pancreatic endocrine and exocrine tumour malignant; neuroendocrine carcinoma NOS; small cell carcinoma NOS

^e^Other morphology includes: enterochromaffin carcinoid; gastrinoma malignant; glucagonoma malignant; insulinoma malignant; mixed pancreatic endocrine and exocrine tumour malignant; pancreatic endocrine tumour malignant; somatostatinoma malignant; tubular carcinoid; and vipoma malignant

**Fig. 1:**
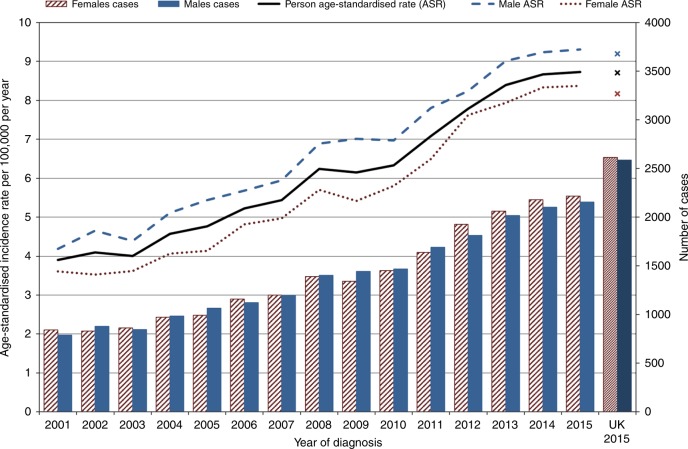
Annual incidence (line graph) and count (bar chart) of NENs diagnosed in England 2001–2015 and the UK 2015 (cross markers)

The 2015 UK annual incidence was 8.7 per 100,000 (8.2 [95% CI, 7.8–8.5] in females and 9.2 in males [95% CI, 8.8–9.6]). It increased steadily, from 3.9 cases per 100,000 in 2001 to 7.9 per 100,000 in 2012 in England, at around 0.4 cases per 100,000 per year (Fig. 1 and Table 2), although it must be noted that these figures are from ICD-O-2 coded data so do not include morphology codes 8013 and 8249.

### Survival

The overall 1YS probability for persons diagnosed with NENs was 74% (Table 2), significantly higher in females 78% than in males 71% (*p* < 0.001 for comparison between sexes). There was a small but significant difference between the least deprived 77% and most deprived 73%. As expected, small cell neuroendocrine carcinoma had the poorest survival probability of all morphologies, 41.4%, whilst NETs had the highest at >90% (inclusive of all sites).

The median survival for oesophageal, prostate and bladder NENs were 5.7 (4.5–7.5), 7.8 (5.8–9.1) and 11.3 (9.9–12.8) months, respectively. Survival for the other sites exceeded 50% at the longest time period so median survival could not be calculated for these.

The 1YS probability for people diagnosed with pulmonary, pancreatic, stomach and small intestine NENs, predominantly well differentiated NETs,  was much higher than for non-NENs at these sites. Conversely, the 1YS probability for people diagnosed with oesophageal, prostate, bladder and to a lesser extent female reproductive organ, colon, and breast NENs, predominantly NECs,was much lower than for non-NENs at these sites (Table 3) and these tumours were at least three times more likely to be diagnosed at stage IV. Around 42% of oesophageal NENs were stage IV and grade 3 tumour pathology with a 1YS probability of 22% (95% CI 16.7–28.5); the predominant morphology at this site was small cell neuroendocrine carcinoma (Table 1). Likewise, 75% of bladder and 74% of prostate NENs were small cell carcinomas or combined small cell carcinomas.

**Table 3:** 1-year survival probability comparison between cancer morphology (NENs vs non-NENs) and association between stage at diagnosis (localised vs metastasized) and cancer morphology for people diagnosed in England 2013–2015

Site	Total number of cases (stage I–IV) (*n*)		1-year overall survival probability (%)		Association between NEN morphology and a diagnosis at stage 4			
	NENs	Non-NENs	NENs (95% CI)	Non-NENs (95% CI)	OR (95% CI) (unadjusted)	*p*-value	OR (95% CI) (age-and sex-adjusted)	*p*-value
Bladder	362	19,859	52.4 (47.4–57.1)	74.9 (74.4–75.5)	3.2 (2.6–4.0)	<0.000	3.2 (2.6–4.1)	<0.000
Breast	51	117,052	82 (68.3–90.2)	96.5 (96.4–96.6)	3.9 (1.9–8.2)	<0.000	3.7 (1.8–7.7)	<0.000
Colon and caecum	458	50,850	67.4 (62.8–71.6)	82.6 (82.3–82.9)	4 (3.3–4.8)	<0.000	3.8 (3.2–4.6)	<0.000
Female reproductive organs	202	40,376	64.7 (57.4–71.1)	89.5 (89.2–89.8)	4.2 (3.2–5.7)	<0.000	5.2 (3.9–7)	<0.000
Oesophagus	296	16,383	35.3 (29.6–41)	51.6 (50.8–52.4)	3.3 (2.6–4.2)	<0.000	3.6 (2.8–4.5)	<0.000
Pancreas	838	8,936	77.6 (74.5–80.3)	31 (29.7–31.5)	0.6 (0.5–0.7)	<0.000	0.5 (0.5–0.7)	<0.000
Prostate	52	98,963	21.1 (11.3–33.0)	95.5 (95.4–95.6)	41.7 (16.6–104.9)	<0.000	45.1 (17.8–113.9)	<0.000
Pulmonary	1965	72,672	74.9 (72.9–76.8)	47.2 (46.8–47.6)	0.4 (0.4–0.5)	<0.000	0.4 (0.4–0.5)	<0.000
Rectum	333	23,498	76.4 (71.4–80.7)	86.8 (86.4–87.3)	1.6 (1.3–2)	<0.000	1.5 (1.2–2.0)	<0.000
Skin (non-melanoma)^a^	876	81,671	74.4 (71.6–77)	92 (91.8–92.2)	ND	ND	ND	ND
Small Intestine	1164	1157	91.2 (89.4–92.7)	61 (58.4–63.1)	0.9 (0.8–1.1)	0.314	0.9 (0.8–1.1)	0.326
Stomach	278	10,938	63.4 (57.3–68.9)	48 (47.4–49.1)	1.0 (0.8–1.3)	0.24	0.9 (0.8–1.2)	0.881

Excluding morphologies neoplasm NOS, carcinoma NOS, tumour cells NOS and basal cell carcinomas

*ND* not determined

^a^non-melanoma skin cancers not staged

Survival decreased more with increasing stage (*p* < 0.001 for comparison between all stages) and grade (*p* < 0.001, for comparison between all grades) combined, identifying the combination as potentially a better prognostic indicator than each variable independently (Supplementary Table 2). This was particularly noticeable for grade 1 and 2 combined vs grade 3 survival probabilities in those with stage IV: lung NENs, 65.1% (56.2–72.6) vs 26.2% (22.5–30), colon NENs, 81.7% (73.1–87.8) vs 21.6% (14.7–29.5); and pancreatic NENs 86.8% (81.4–90.8) vs 41.1% (34.6–47.4).

Univariable Cox regression analysis determined that the hazard of death increased by 4% (HR 1.04 95% CI [1.03–1.06]) for each increase in deprivation of 1 quintile; 21% (HR 1.21 95% CI [1.19–1.22]) for every 5 year increase in age; and 35% (HR 1.35 95% CI [1.32–1.38]) with each one increment increase in stage at diagnosis. Risk decreased by 5% (HR 0.95 95% CI [0.92–0.98]) for each year of diagnosis between 2013 and 2015.

Multivariable Cox analysis adjusting for predictor variables sex, age, stage, site, morphology, grade, deprivation and year of diagnosis on mortality (Table 2) determined the hazard of death was up to 18% lower for women than men; those aged over 75 years three times that of those aged under 54 years; and those with stage IV NENs more than twice that of those with stage III NENs. Some sites were associated with increased hazard, the hazard in those with gallbladder, anal and oesophageal NENs was similar to that of those with secondary tumours (HR 3.2 95% CI [2.5–4.1]) (Supplementary Table 1). Those who lived in the most deprived areas had up to a 41% increase in hazard of death when compared with the least deprived.

### Mortality

SMRs were used to measure survival relative to the general population (Table 2). Deaths occurred nearly four times more frequently in those diagnosed with NENs; varying from nearly three times more in those aged over 75 years to 10 times more in those aged 55–64 years at diagnosis, and with the same frequency as those in the general public for those with stage I to 10 times more in those with stage IV cancer.

## Discussion

### Incidence

The incidence of NENs appears to be rising in this and other international studies.^14,15^ This rise may be real, or may be an artefact of the use of diagnostic imaging^15^ with improved sensitivity, and increased clinical vigilance resulting in incidental detection of asymptomatic lesions.^14^ Also, in this study the upward trend in incidence between 2013, the beginning of ICD-O-3 coding in the UK and 2015 is less pronounced than that seen previously. We need future studies using a wider timeframe to determine whether the incidence of NENs is still rising or beginning to plateau.

In the UK, the incidence of NENs in the rectum ranked after lung, small intestine, appendix, pancreas, stomach and colon and caecum combined, in comparison with the most recent international study, in the USA,^15^ where rectum was the 3rd most common after lung and small intestine, with appendix last. In the USA, colorectal screening starts at 50 years old^16^ compared with 55 years in the UK and could be improving detection; appendiceal NETs only became reportable in the USA from January 2015 (SEER personal communication), explaining the comparatively lower incidence.

### Survival and mortality

Multivariable analysis identified sex, age, site, stage, cell morphology and deprivation to be independently associated with mortality. Survival probabilities for NENs in the UK had similar trends to previous international studies with male sex, increasing age, stage, and grade and decreasing socioeconomic status associated with a poorer outcome.^13,15^ The much reduced 1YS and mortality in metastatic tumours (stage IV) when compared with localised tumours (stage I–III) was particularly notable and in-line with a study of “all cancers” diagnosed in the UK,^17^ warranting further measures to ensure that the early diagnosis of NENs is a priority. People diagnosed with oesophageal, bladder, prostate, and female reproductive NENs had predominantly small cell carcinoma morphology, significantly poorer outcomes consistent with the results of previous smaller studies and case series,^18–21^ and were more likely to be diagnosed at stage IV. Some morphologies exhibited poorer survival probabilities than expected. This was the first large series study of MANEC reported and demonstrated a survival probability of 84% in comparison with the 95% previously reported.^22^ These findings highlight the need for the development of sub-specialist services to match the clinical need in the groups which have poorer outcomes.

### Limitations

Tumours with uncertain behaviour (behaviour code 1) are not consistently captured by UK cancer registries because they have historically been deemed “benign” and were not included. This means that many small and indolent NETs of the stomach, rectum, appendix and pancreas may not have been included in historical data. There is an under representation of type 1 gastric NETs and stage I and II rectal NETs—that all have excellent 5- and 10-year survivals. This under representation may be significantly skewing the survival statistics presented in this study. Also, the high incidence of G3 NECs in this study, particularly those in the lung, are not representative of previous findings. In the lung, G3 NEC is used when there has been insufficient information to classify the tumour as either small cell carcinoma or large cell neuroendocrine carcinoma. Thus, the high incidence of G3 NEC in the lung is likely to represent small cell carcinoma, large cell neuroendocrine carcinomas and mixed tumours. This misclassification of G3 NECs could also be skewing the survival analysis. This is a caveat for ongoing classification recommendations for the purposes of analysis and audit. In the future, many of these tumours will be more consistently captured with improvements in WHO terminology.

The completeness of stage was only 61% for this cohort but is improving year on year. ICD-O-3 coding was implemented in 95% of the UK in 2013 (the exception Wales in 2016), prior to this morphology codes 8013 and 8249 were not available, many of these tumours coded carcinoma or adenocarcinoma NOS (not otherwise specified). Therefore, our incidence counts are underestimates, and survival analysis limited to only 1-year.

We calculated that the missing tumours from the Welsh cohort would equate to approximately 62 from common sites. Also, an audit of the English data using data collected by the NET Centre of Excellence at King’s College Hospital found 14.6% of the tumours captured were not captured by NCRAS, many behaviour 1. This would suggest the true incidence of NENs could be as high as 10 per 100,000 per year.

The capture of NENs irrespective of behaviour code, ICD-O-3 coding in Wales from 2016 and improvements in data completeness will resolve the afore mentioned issues in the future.

We were unable to calculate disease-specific survival and mortality due to death registrations by ICD-10 coding. However, recently it has become possible to link morphology to death registrations which will allow this in future studies.

## Conclusion

This is the first population-based epidemiological study of NENs diagnosed in the UK, the results of which will be of value in future service planning. We determined that outcomes varied greatly between sites and morphologies. Comparison of NENs with non-NENs at the same site also found disparities. These differing outcomes between patient groups have a direct relationship with the healthcare resource required to manage them; and highlight the need for NENs to be seen at Centres of Excellence. Earlier diagnosis is necessary, and the identification of more diagnostic markers for NENs, particularly for extrapulmonary small cell carcinomas, required.

Future studies will allow 3- and 5-year survival estimates, and the availability of treatment data will allow more in-depth studies, and we are developing ways of collecting more detailed imaging, biochemistry, screening and symptom data which ultimately will be linked to the current cancer registry data. Pathologists have been instructed to include small benign pancreatic NETs as malignant as per WHO terminology and detail Ki67 on all specimen. Ki67 has not been routinely captured by the UK cancer registries to date, however this will change in mid-2020 with the implementation of version 9 of the Cancer Outcome and Services Data set (COSD), the national standard for reporting cancer in the NHS in England, in which Ki67 will be a required field and so captured by the English registry. Although the UK cancer registries do not currently hold complete data on MEN-1 diagnosis, the recent addition of genetic data means that this may also be available for future studies.

## Supplementary information

Supplementary Table 1: NEN incidence, 1-year survival probability, univariable and multivariable analysis, and mortality in UK (online only)

Supplementary Table 2: 1-year survival probability (%) for pulmonary, small intestinal, appendiceal, pancreatic, stomach, rectal, and oesophageal NENs diagnosed in the UK between 2013 and 2015, by grade and stage (online only)

## Data Availability

Supplementary datasets cited have been made available as supplementary material. Other data for this study is available on request from PHE NCRAS, NHS National Services Scotland, Public Health Wales Welsh Cancer Intelligence & Surveillance Unit and Public Health Agency Queen’s University Belfast.

## References

[1] Kunz PL, Reidy-Lagunes D, Anthony LB, Bertino EM, Brendtro K, Chan JA (2013). Consensus guidelines for the management and treatment of neuroendocrine tumors. Pancreas.

[2] Singh S, Granberg D, Wolin E, Warner R, Sissons M, Kolarova T (2016). Patient-reported burden of a neuroendocrine tumor (NET) diagnosis: results from the first global survey of patients with NETs. J. Glob. Oncol..

[3] Fritz A., Percy C., Jack A., Shanmugaratnam K., Sobin L., Max Parkin D. (eds.). *International Classification of Diseases for Oncology*, 3rd edn, first revision. (World Health Organization, Geneva, 2013).

[4] Sobin L. H., Gospodarowicz M. K., Wittekind C. *International Union Against Cancer TNM Classification of Malignant Tumors*, 7th edn. (John Wiley & Sons, Hoboken, 2011).

[5] Edge S. E., Byrd D. R., Carducci M. A., Compton C. C., Fritz A. G., Greene F. L. et al. *American Joint Committee on Cancer (AJCC) TNM staging for NETs. AJCC Cancer Staging Manual*, 7th edn. (Springer, New York, 2010).

[6] Kloppel G, Couvelard A, Perren A, Komminoth P, McNicol AM, Nilsson O (2009). ENETS consensus guidelines for the standards of care in neuroendocrine tumors: towards a standardized approach to the diagnosis of gastroenteropancreatic neuroendocrine tumors and their prognostic stratification. Neuroendocrinology.

[7] Bosman F. T., Carneiro F., Hruban R. H., Theise, N. D. *World Health Organisation classification of tumours of the digestive system*. 4th edn. (IARC, Lyon, 2010)

[8] Rindi G, Kloppel G, Alhman H, Caplin M, Couvelard A, de Herder WW (2006). TNM staging of foregut (neuro)endocrine tumors: a consensus proposal including a grading system. Virchows Arch..

[9] Rindi G, Kloppel G, Couvelard A, Komminoth P, Körner M, Lopes JM (2007). TNM staging of midgut and hindgut (neuro) endocrine tumors: a consensus proposal including a grading system. Virchows Arch..

[10] Office for National Statistics, (2016). *Mid-2015 Population estimates for lower layer super output areas in England and Wales by single year of age and sex—supporting information (SAPE18DT1) annual small area population estimates.* (Office for National Statistics (ONS), London, 2016).

[11] Cronin KA, Feuer EJ (2000). Cumulative cause-specific mortality for cancer patients in the presence of other causes: a crude analogue of relative survival. Stat. Med.

[12] Office for National Statistics. *National Records of Scotland, Northern Ireland Statistics and Research Agency. Death registrations by single year of age, United Kingdom, 1974–2017*. pp. 2018. (ONS, London, 2018).

[13] Yao JC, Hassan M, Phan A, Dagohoy C, Leary C, Mares JE (2008). One hundred years after “carcinoid”: epidemiology of and prognostic factors for neuroendocrine tumors in 35,825 cases in the United States. J. Clin. Oncol..

[14] Hallet J, Law CH, Cukier M, Saskin R, Liu N, Singh S (2015). Exploring the rising incidence of neuroendocrine tumors: a population-based analysis of epidemiology, metastatic presentation, and outcomes. Cancer.

[15] Dasari A, Shen C, Halperin D, Zhao B, Zhou S, Xu Y (2017). Trends in the Incidence, prevalence, and survival outcomes in patients with neuroendocrine tumors in the United States. JAMA Oncol..

[16] Wolf AMD, Fontham ETH, Church TR, Flowers CR, Guerra CE, LaMonte SJ (2018). Colorectal cancer screening for average-risk adults: 2018 guideline update from the American Cancer Society. CA Cancer J. Clin..

[17] Bannister, N., Broggio, J. (eds.). *Cancer survival by stage at diagnosis for England (experimental statistics): adults diagnosed 2012, 2013 and 2014 and followed up to 2015.* (Office for National Statistics, London, 2016).

[18] Hudson E, Powell J, Mukherjee S, Crosby TD, Brewster AE, Maughan TS (2007). Small cell oesophageal carcinoma: an institutional experience and review of the literature. Br. J. Cancer.

[19] Emmett M, Gildea C (2018). Cervical cancer—does the morphological subtype affect survival rates?. J. Obstet. Gynaecol..

[20] Watson GA, Ahmed Y, Picardo S, Chew S, Cobbe S, Mahony C (2018). Unusual sites of high-grade neuroendocrine carcinomas: a case series and review of the literature. Am. J. Case Rep..

[21] Eltawil KM, Gustafsson BI, Kidd M, Modlin IM (2010). Neuroendocrine tumors of the gallbladder: an evaluation and reassessment of management strategy. J. Clin. Gastroenterol..

[22] Brathwaite S, Rock J, Yearsley MM, Bekaii-Saab T, Wei L, Frankel WL (2016). Mixed adeno-neuroendocrine carcinoma: an aggressive clinical entity. Ann. Surg. Oncol..

